# Influence of the tilt angle of Percutaneous Aortic Prosthesis on Velocity and Shear Stress Fields

**DOI:** 10.5935/abc.20170115

**Published:** 2017-09

**Authors:** Bruno Alvares de Azevedo Gomes, Gabriel Cordeiro Camargo, Jorge Roberto Lopes dos Santos, Luis Fernando Alzuguir Azevedo, Ângela Ourivio Nieckele, Aristarco Gonçalves Siqueira-Filho, Glaucia Maria Moraes de Oliveira

**Affiliations:** 1 Programa de Pós Graduação em Cardiologia - Universidade Federal do Rio de Janeiro, Rio de Janeiro, RJ - Brazil; 2 Pontifícia Universidade Católica do Rio de Janeiro (PUC-Rio), Rio de Janeiro, RJ - Brazil; 3 Instituto Nacional de Cardiologia, INC/MS, Rio de Janeiro, RJ – Brazil

**Keywords:** Heart Valve Prosthesis Implantation, Regional Blood Flow, Hemodynamics, Shear Stress

## Abstract

**Background:**

Due to the nature of the percutaneous prosthesis deployment process, a variation in its final position is expected. Prosthetic valve placement will define the spatial location of its effective orifice in relation to the aortic annulus. The blood flow pattern in the ascending aorta is related to the aortic remodeling process, and depends on the spatial location of the effective orifice. The hemodynamic effect of small variations in the angle of inclination of the effective orifice has not been studied in detail.

**Objective:**

To implement an *in vitro* simulation to characterize the hydrodynamic blood flow pattern associated with small variations in the effective orifice inclination.

**Methods:**

A three-dimensional aortic phantom was constructed, reproducing the anatomy of one patient submitted to percutaneous aortic valve implantation. Flow analysis was performed by use of the Particle Image Velocimetry technique. The flow pattern in the ascending aorta was characterized for six flow rate levels. In addition, six angles of inclination of the effective orifice were assessed.

**Results:**

The effective orifice at the -4º and -2º angles directed the main flow towards the anterior wall of the aortic model, inducing asymmetric and high shear stress in that region. However, the effective orifice at the +3º and +5º angles mimics the physiological pattern, centralizing the main flow and promoting a symmetric distribution of shear stress.

**Conclusion:**

The measurements performed suggest that small changes in the angle of inclination of the percutaneous prosthesis aid in the generation of a physiological hemodynamic pattern, and can contribute to reduce aortic remodeling.

## Introduction

Transcatheter Aortic Valve Implantation (TAVI) has been introduced by Cribier et al.^1^ as an alternative to treat individuals with severe aortic valve stenosis at high surgical risk. With the development of new systems of percutaneous heart valve implantation, the use of TAVI for patients at intermediate surgical risk has been a worldwide trend.^2-4^ Because of the nature of the implantation procedure, a variation in prosthetic valve placement is expected.^5^ In addition, eccentric calcifications in the aortic annulus can influence the final orientation of the valve prosthesis. Valve placement will define the spatial location of its effective orifice in relation to the aortic annulus, and will determine the likelihood of generating eccentric flow in the vascular lumen.^6^

Several studies have shown that the anatomical characteristics of the aortic root influence blood flow in the ascending aorta.^7^ In addition, the changes in blood flow pattern after TAVI represent an important aspect that has not been studied in details.^5^ Studies suggest that eccentric blood flow is related to the aortic remodeling process, such as dilatation and aneurysmal formations.^8-10^

*In vitro* simulations that preserve the anatomy of the aorta (patient-specific) can contribute to a better understanding of the blood flow changes produced by variations in the effective orifice inclination. Contrary to *in vivo* studies, *in vitro* simulations enable proper control of flow geometry and contour conditions, providing a systematic assessment of the blood flow response to valve placement variations.

So far, only one study^11^ using flow-sensitive cardiovascular magnetic resonance imaging (4D flow MRI)^12^ has reproduced the anatomy of the aorta of a patient and has assessed the changes in blood flow produced by variations in the effective orifice inclination. The objective of the present study was to implement an *in vitro* simulation to characterize the hydrodynamic pattern of blood flow associated with small variations in the effective orifice inclination.

## Methods

This is a descriptive study of *in vitro* simulation of blood flow in a three-dimensional (3D) aortic model. For that purpose, a vascular phantom was constructed based on the tomographic angiography of the aorta of one patient submitted to TAVI. The present study was approved by the Research Ethics Committee of the institution. The patient was a 77-year-old male with severe degenerative aortic stenosis, mild ventricular dysfunction, and New York Heart Association functional class III.

**Figure 1:**
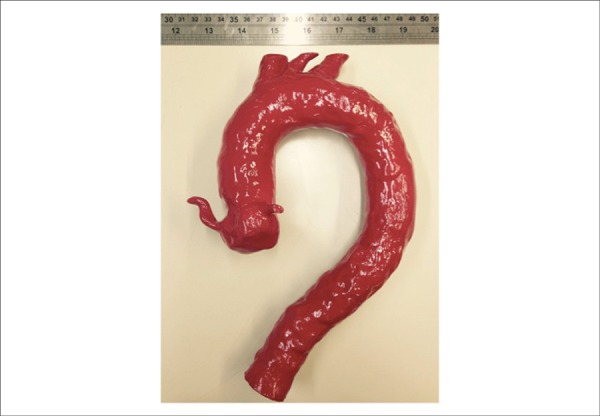
Three-dimensional aortic model. Model in ABS-M30 Affordable FDM thermoplastic material (Stratasys, USA).

Tomographic angiography of the aorta was performed with a Somatom Sensation 64-channel tomography device (Siemens, Germany). Tomographic slices from the aortic annulus to the distal segment of the thoracic aorta were selected. The DICOM images were transferred to the Mimics software (Materialise, Belgium) to implement the segmentation of the aortic region of interest. After the segmentation process, the digital file was exported to.*STL* (stereolithography) format to perform the 3D printing, with the Stratasys Fortus 400 mc Systems equipament (Stratasys, USA), using the ABS-M30 Affordable FDM thermoplastic material (Stratasys, USA). The 3D model was printed in a real scale, and its dimensions were confirmed via measurements taken on the tomographic angiography of the aorta (Figure 1).

The 3D aortic model was used to build a silicone phantom, with which the *in vitro* simulation of blood flow was implemented. The 3D model was positioned in a rectangular reservoire, built with plexiglass plaques. Liquid silicone elastomer was added to that reservoire, involving the aortic model. After 24 hours, the silicone elastomer was solid, allowing the extraction of the 3D model. A longitudinal cut was performed in the lateral walls of the elastomer block, dividing it into two halves. Then, the 3D model was removed from the silicone phantom, and the two halves were reconnected. To maintain proper alignment and to preserve the original anatomy of the aorta, five metallic rods, crossing the entire set, were used to guide the reassembly of the phantom. After reuniting the two halves of the phantom, connectors of the hydraulic circuit allowed the test solution to flow through the silicone model.

The Sylgard 184 silicone elastomer (Dow Corning, USA) was chosen because of its optical properties, and an image technique with laser was used to measure the flow patterns. That silicone has a refraction index (n = 1,417) close to that of the test solution chosen for the assays, an aqueous mixture of glycerine (60% glycerine, n = 1,420).^13^ The test solution was drained into a closed circuit through the hydraulic instalation, boosted by a constant-volume pump, NEMO 4501140 (NETZSCH of BRASIL, Brazil). The flow rate was adjusted by controlling the frequency of pump rotation, using a frequency inversor CFW 08 (WEG, Brazil).

The flow was directed to the aortic phantom, with inflow in the vascular lumen occurring in the position equivalent to the aortic annulus, where a nozzle was connected to the phantom, representing the aortic prosthesis with full opening of its leaflets. The inner area of that nozzle measured 1.5 cm^2^, based on the effective orifice of the patient’s prosthesis, obtained via transthoracic echocardiography. The aortic phantom had the following outflow points: brachiocephalic trunk, left common carotid artery, left subclavian artery, and thoracic aorta.

The Particle Image Velocimetry (PIV) technique was chosen for flow analysis.^14^ The particles that served as flow tracers were constituted by silver-coated hollow glass espheres of approximately 13 µm of diameter, and were addeded to the aqueous glycerine solution. A dual-cavity laser (BIG SKY Nd: YAG, 120 mJ, Quantel, USA) was the illumination source used, generating a 0.5-mm-thick light plane. A digital camera (PIVCAM 10-30, TSI, USA) captured synchronized images of the particles in the region between the aortic annulus and the middle ascending aortic segment. For each implemented hydrodynamic state, 3000 images of the tracers were captured, producing 1500 instantaneous velocity fields. The mean velocity and shear rate fields were calculated based on those instantaneous fields. Cross-correlation was the process used to determine the displacement of the tracers, by use of the INSIGHT 3G software (TSI, USA). Each velocity vector was obtained for an area of 32x32 pixels in the image, corresponding to a 2x2-mm resolution in the real flow.^14^

The PIV technique produced two-dimensional velocity fields. To have a 3D characterization of the aortic flow, the measurements were taken in four different planes. The central measuring plane was placed as to coincide with the central line of the effective orifice, crossing the right coronary ostium, and encompassing the main flow inside the aortic phantom. In addition, the velocity measurements were taken in three other planes, 4 mm apart from each other. Two of those planes were placed towards the dorsal region and one was placed towards the ventral region.

Because of the rapid blood flow acceleration at the beginning of the ventricular systole, it was hypothesized that significant changes in shear stress occur during that period.^15^ Thus, the present study was designed to characterize the flow in the initial third of the ventricular systole. For that purpose, the following values of continuous flow were used: 0.8; 1.6; 2.6; 3.3; 4.0 and 5.3 liters per minute (L/min). Considering the properties of the test solution and the inner diameter of the effective orifice, the Reynolds numbers corresponding to each flow level were 195, 390, 630, 800, 970 and 1285, respectively.

**Figure 2:**
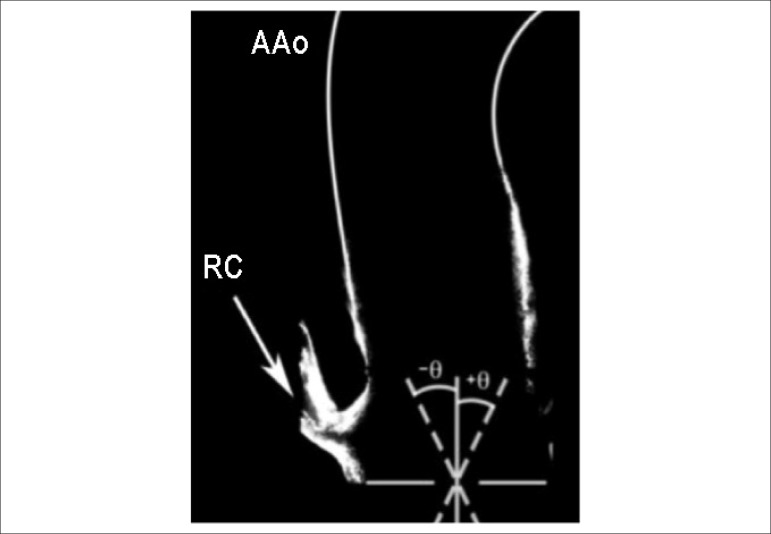
Angle of inclination of the effective orifice. AAo: ascending aorta; RC: right coronary artery; θ: angle of inclination.

The variation in the inclination of the effective orifice could be assessed by building a spindle inclination mechanism, comprising a threaded rod coupled to a 0-25-mm micrometer (Mitutoyo, Japan). In one extremity of that rod, a joint allowed coupling the spindle inclination mechanism to the entrance nozzle that was connected to the aortic phantom. When a translation movement was imposed to the spindle inclination mechanism, there would be a change in the effective orifice inclination. For flows of 2.6 and 3.3 L/min, the following angles of inclination were implemented: -4º, -2º, 0º, +1º, +3º, and +5º. The zero angle of inclination corresponded to the coincidence of the central line of the effective orifice with the central line of the aortic annulus. The negative angles tilted the main flow towards the right coronary ostium, while the positive angles tilted the main flow towards the posterior wall (Figure 2).

## Results

**Figure 3:**
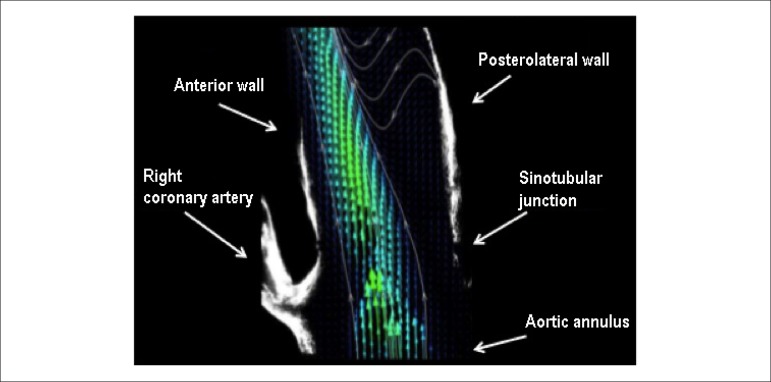
Velocity field measured inside the aortic phantom.

The results of the analysis of the flow between the aortic annulus and the middle ascending aortic segment are now presented. Because the software used to implement the PIV technique provides no information on the physical boundaries limiting the flow, an image of the vascular model was overlapped with a typical velocity field. Figure 3 shows the anatomical structures close to the flow area in the aortic phantom.

The following results include the velocity and shear rate fields for the four measurement planes described. For each plane, the results are presented for six flow rate values, ranging from 0.8 to 5.3 L/min. Subsequently, for the central plane, the results will explore the effect of the variation in the effective orifice tilt angle.

### Velocity field

**Figure 4:**
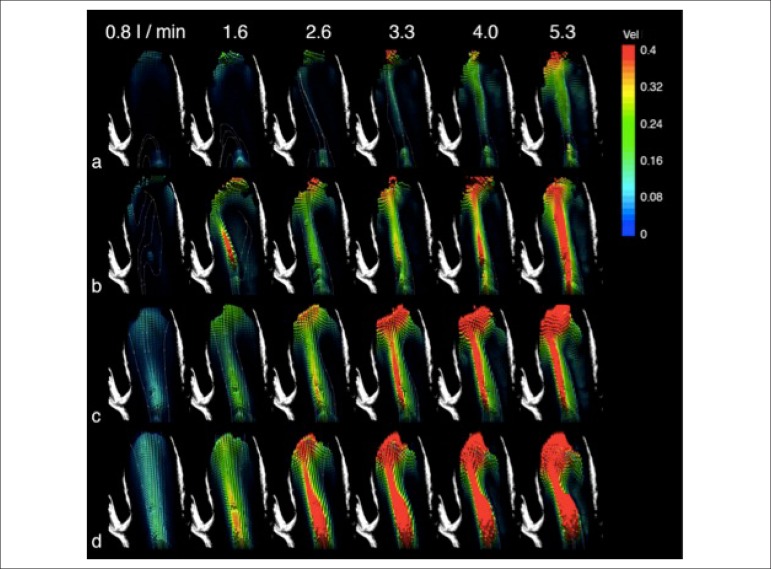
Velocity fields in the measurement planes. Velocity fields in the (a) 4-mm ventral, (b) central, (c) 4-mm dorsal, and (d) 8-mm dorsal planes. Velocity magnitude in meters per second.

Figure 4 shows the results for the mean velocity fields measured in the aortic phantom for four different planes. For each plane, six flow rate values are shown. The velocity vectors are colored according to their magnitude (meters per second - m/s), based on the scale at the right side of the figure.

The experimental tests represented the initial third of the ventricular systole, reaching maximum instantaneous velocities of approximately 1.2 m/s. For all velocity fields, the color scale of magnitude was maintained fixed, aiming at comparig between the different hydrodynamic states. Although the measures reached 1.2 m/s, the color scale was adjusted between 0 and 0.4 m/s, enabling the comparison between different cases, because, in the ventral plane, the low velocity values predominated. For each plane of measurement, one qualitative analysis of flow is shown.

**4-mm ventral plane.** For the flow rates of 2.6 and 3.3 L/min, the flow is directed towards the anterior wall. As the flow rate increases to 4.0 and 5.3 L/min, a larger part of the main jet acquires a centralized configuration, reaching a velocity of 0.4 m/s, at a flow rate of 5.3 L/min (Figure 4a).

**Central plane.** In this plane, the main jet is well defined to the flow rate of 1.6 L/min, and markedly inclined towards the anterior wall. As the flow rate increases, the main jet widens, showing a mild trend towards flow centralization (Figure 4b).

**4-mm dorsal plane.** In the first dorsal plane, the main jet is well defined as a dominant flow structure. From the flow rate of 3.3 L/min, maximum velocity is observed from the sinotubular junction to the middle ascending aortic segment. As the flow rate increases to 4.0 and 5.3 L/min, the maximum velocity region increases, seen as the dominance of the red color region (Figure 4c).

**8-mm dorsal plane.** In this plane, a continuous maximum velocity region is observed for the flow rate of 2.6 L/min, occupying the area from the sinotubular junction to the middle ascending aortic segment. In this plane, for all flow rate levels, left inclination towards the anterior wall is seen. The analysis of the velocity field for the flow rate of 5.3 L/min clearly shows that the main jet falls on the anterior wall, as seen by the large maximum velocity area (Figure 4d).

### Shear rate

**Figure 5:**
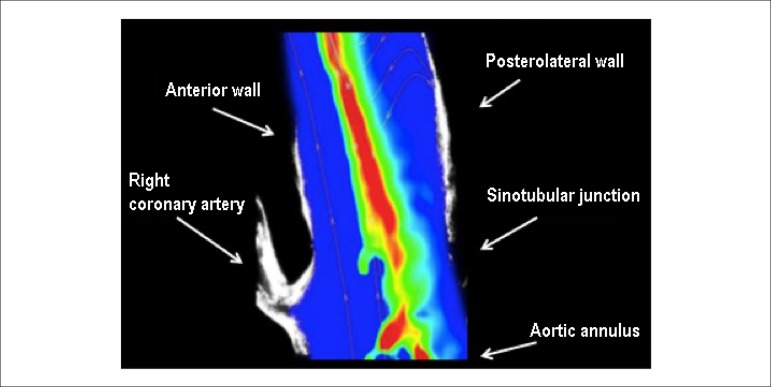
Shear rate inside the aortic phantom.

The shear rate fields, calculated from the velocity fields shown in Figure 4, are now presented. As previously performed for the velocity fields, in Figure 5, an image of the vascular phantom was overlapped with a shear rate field to make the interpretation of results easier.

**Figure 6:**
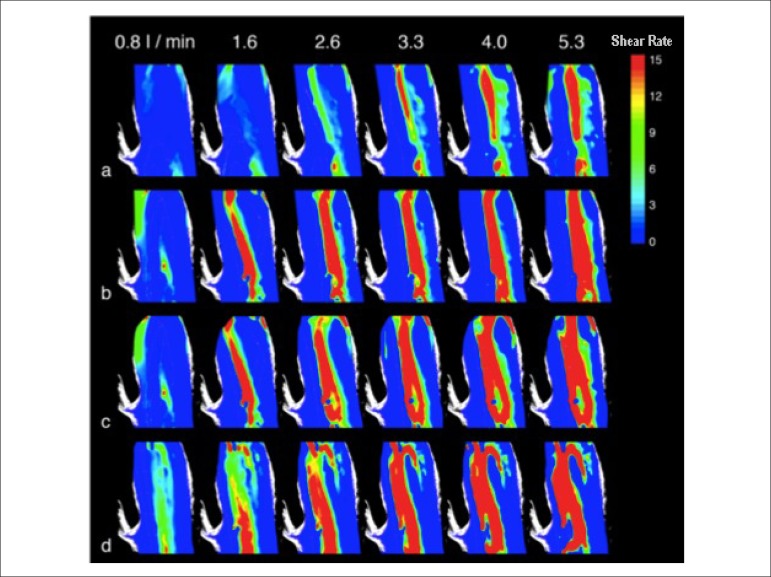
Shear rate in the measurement planes. Shear rate in the (a) 4-mm ventral, (b) central, (c) 4-mm dorsal, and (d) 8-mm dorsal planes. Shear rate magnitude in s^-1^.

The results of shear rate are shown in Figure 6, being exhibited for the same planes and flow rates of the velocity fields. The color scale in the figure represents the shear rate magnitude, ranging from 0 to 15 s^-1^.

**4-mm ventral plane.** In the ventral plane, a high shear rate region is identified at the flow rate of 2.6 L/min. At 3.3 L/min, maximum shear rate occurs, as indicated by the red band, which is elongated and leans towards the anterior wall. At higher flow rates, 4.0 and 5.3 L/min, the red band extends from the top to the bottom of the image. High shear rate regions are found close to the effective orifice, in the lower part of the figure (Figure 6a).

**Central plane.** In this plane, maximum shear is already identified, even in an incipient manner, at the flow rate of 0.8 L/min. From the flow rate of 1.6 L/min, the maximum shear stress band occupies the entire extension of the images. At subsequent flow rates, progressive widening of that area is seen (Figure 6b).

**4-mm dorsal plane.** From the flow rate of 2.6 L/min, maximum shear stress bends towards the anterior wall. In addition, expressive widening of that band is observed as the flow rate increases. Despite the pattern of inclination to the left, it is worth noting the presence of a small sector with maximum shear stress at the right upper part of the images, beginning at the flow rate of 2.6 L/min (Figure 6c).

**8-mm dorsal plane.** In this plane, widening of the maximum shear region is also seen from the flow rate of 2.6 L/min. The inclination of the high shear region towards the left upper part of the images remains, showing a preferential direction towards the anterior wall (Figure 6d).

### Influence of the angle of inclination of the effective orifice

The influence of the inclination of the effective orifice on flow characteristics was assessed by use of measurements taken in the central plane, for the flow rates of 2.6 and 3.3 L/min. The angles of inclination ranged from -4º to +5º, as shown in Figure 2.

### Velocity and shear rate fields

**Figure 7:**
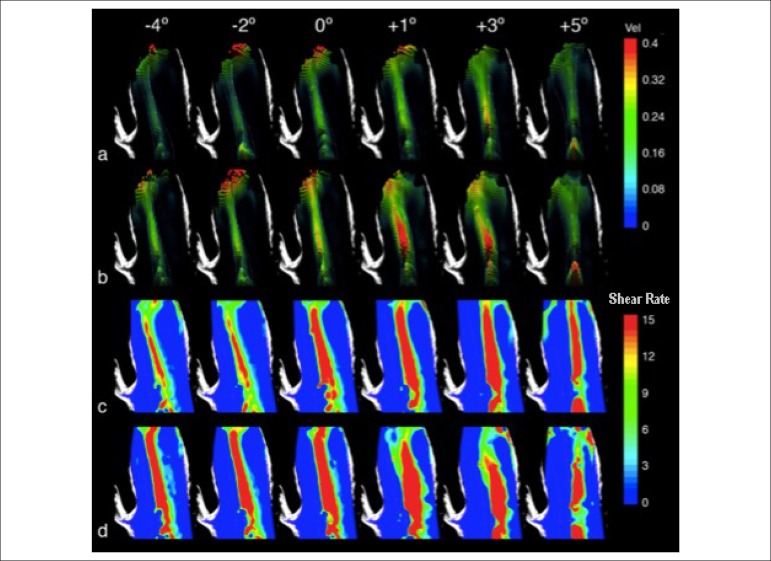
Velocity and shear rate fields for (a, c) 2.6 L/min and (b, d) 3.3 L/min. Inclination of the effective orifice: -4º, -2º, 0º, +1º, +3º, and +5º.

Figure 7 shows the influence of the angle of inclination of the effective orifice on the velocity and shear rate fields. For 2.6 L/min, at a zero angle of inclination, the main flow was directed to the left, reaching the anterior wall in the middle ascending aortic segment. When the effective orifice was placed at negative inclinations (-4º and -2º), that flow eccentricity was exacerbated. For small positive inclinations (+1º, +3º and +5º), a trend towards flow centralization was observed. With that mild change in inclination, the main flow is directed to the posterolateral wall (Figure 7a).

At 3.3 L/min, the velocity fields show that, for the negative angles, the main jet decreased its width as compared to that at zero angle of inclination. The negative angles show similar velocity magnitudes, with a left inclination. When the inclination reaches positive values, a trend towards centralization of the main jet is observed. Thus, higher velocity values appear inside the jet, as seen by the red colored regions. The velocity patterns are similar at the angles +1º and +3º, maintaining a left inclination in the upper half of the image. The +5º angle of inclination shows a more significant centralization of the main jet (Figure 7b).

Figures 7c and 7d show the results for shear rate. At 2.6 L/min, for the negative inclinations, the red color bands are narrower as compared to those of other angle positions. For positive inclinations, maximum shear area widening and centralization are seen (+3º and +5º). For the +5º inclination, the maximum shear region is maintained close to the central line of the aortic model (Figure 7c).

The analysis of the shear rate results at the 3.3-L/min flow rate indicates that, for negative inclinations, maximum shear shows a left inclination. In the positive inclinations of +1º and +3º, maximum shear is located in the central region. Proximity of the high shear region to the posterolateral wall is observed at the +5º angle, because the red band occupies the right side of the image (Figure 7d).

## Discussion

In the present study, an *in vitro* simulation was performed to characterize the hydrodynamic pattern of blood flow during ventricular systole in a 3D aortic model representing the anatomy of a patient submitted to TAVI. In addition, by use of velocity and shear rate fields, we identified flow changes due to six variations in the angle of inclination of the effective orifice.

The optimization of percutaneous valve prosthesis implantation, in addition to its placement according to the patient’s native flow pattern, can be a means of ensuring its best performance after TAVI.^16,17^ The generation of a blood flow with a hemodynamic pattern closer to the physiological one can have a beneficial impact on the patients’ survival.^18,19^

The qualitative analysis of the velocity and shear rate fields for each plane analysed in the present study clearly shows the 3D nature of the flow inside the aortic model. These data stress the importance of using realistic models of aorta geometry, in addition to the limitations of *in vitro* studies that represent the aorta using circular and axisymmetric models.^5^

Groves et al.^5^ have studied the effect of the variations in the axial placement of percutaneous prostheses. The aorta was represented with an plexiglass circular tube of 30-mm inner diameter. For a 4-L/min flow rate, only the 5-mm displacement below the aortic annulus resulted in low shear stress values, in addition to a symmetrical distribution.^5^ In the present study, the effective orifice remained positioned less than 5 mm from the aortic annulus. However, because our model preserved the aorta anatomy, the results differed with respect to the planes assessed, which were not axisymmetric as in the study by Groves et al.^5^

Groves et al.^5^ and Wilton et al.^20^ have considered the hypothesis that high shear stress, in addition to its asymmetric distribution, would be related to aortic dilatation and a higher chance of dissection.^5,10,20,21^ Based on that hypothesis, optimizing the axial placement and the effective orifice inclination would be desirable, so that symmetric and low-magnitude shear stress distribution would be obtained. An axial placement of less than 5 mm from the aortic annulus associated with positive angles of inclination would be suggested in this particular case. In addition, Groves et al.^5^ have reiterated that high shear stress levels downstream the prosthesis could contribute to reduce its durability, because of higher mechanical stress, emphasizing the importance of optimizing the prosthesis placement.

Trauzeddel et al.^7^ have assessed post-TAVI ascending aortic blood flow patterns, which were compared to those of patients submitted to conventional stented aortic valve replacement (AVR) and those of healthy individuals. That study showed that both TAVI and AVR resulted in maximum shear stress values in the right anterior wall, while minimum shear stress values were found in the left posterior wall. Healthy individuals, however, showed physiological central blood flow and a symmetric distribution of shear stress along the aortic circumference.^7^ The maximum shear stress distribution in the anterior wall of patients submitted to TAVI and AVR coincides with the results of the *in vitro* simulation of the present study for the negative angles of inclination of the effective orifice. In the experimental model, that angle of inclination could be modified in the search for a configuration that produced a central blood flow pattern. As the results presented indicate, a decrease in shear stress in the anterior wall was obtained with small positive inclinations. For example, at the +3º angle, the maximum shear stress region remained restricted to the central portion of the aortic phantom. When the angle of inclination was increased to +5º, the maximum shear stress region approached the posterolateral wall.

The present analysis was limited to the anatomic findings of one patient. This simulation, however, represented a real 3D anatomy, providing a significant advance in relation to the circular and axisymmetric models used in previous studies.^5,6^

In the present study, only the initial third of the ventricular systole was represented. However, the highest prevalence of high shear stress values in the ascending aorta is known to occur during the systole. In addition, because that period of the cardiac cycle is characterized by sudden changes in velocity, a rapid variation in shear stress values is also expected.^15^ In this study, a segment of pulsatile blood flow, more precisely the initial third of the ventricular systole, was represented by six different continuous blood flow levels. With this approach, some structures of secondary blood flow might not have been captured. However, in cardiovascular science, the various stages of pulsatile blood flow are commonly modelled with an increasing sequence of continuous blood flow values.^11,22^

Based on these findings, projects of new prostheses, with the ability to change the angle of inclination of the effective orifice, can be proposed, enabling the generation of a centralized blood flow in the ascending aorta, mimicking a physiological hemodynamic pattern.

## Conclusion

The present study evidenced the 3D character of the blood flow pattern inside the vascular phantom, and identified a range of optimized values for the angle of inclination of the effective orifice. For small positive inclinations, a physiological centralized blood flow was obtained in the middle ascending aortic segment, eliminating the high mechanical shear stress values in the anterior wall, which prevailed in the negative inclinations of the effective orifice (-4º and -2º). In the placements with positive inclinations, the regions with high shear stress levels were maintained close to the central line of the vascular phantom.
